# SAXS and stability studies of iron-induced oligomers of bacterial frataxin CyaY

**DOI:** 10.1371/journal.pone.0184961

**Published:** 2017-09-20

**Authors:** Mostafa Fekry, Wessen Alshokry, Przemysław Grela, Marek Tchórzewski, Eva-Christina Ahlgren, Christopher A. Söderberg, Oleksandr Gakh, Grazia Isaya, Salam Al-Karadaghi

**Affiliations:** 1 Center for Molecular Protein Science, Department of Biochemistry and Structural Biology, Lund University, Lund, Sweden; 2 Biophysics Department, Faculty of Science, Cairo University, Giza, Egypt; 3 Department of Molecular Biology, Maria Curie-Skłodowska University, Lublin, Poland; 4 MAX IV Laboratory, Lund University, Lund, Sweden; 5 Departments of Pediatric and Adolescent Medicine and Biochemistry and Molecular Biology, Mayo Clinic, College of Medicine, Rochester, Minnesota, United States of America; Sant Joan de Déu Children's Hospital, SPAIN

## Abstract

Frataxin is a highly conserved protein found in both prokaryotes and eukaryotes. It is involved in several central functions in cells, which include iron delivery to biochemical processes, such as heme synthesis, assembly of iron-sulfur clusters (ISC), storage of surplus iron in conditions of iron overload, and repair of ISC in aconitase. Frataxin from different organisms has been shown to undergo iron-dependent oligomerization. At least two different classes of oligomers, with different modes of oligomer packing and stabilization, have been identified. Here, we continue our efforts to explore the factors that control the oligomerization of frataxin from different organisms, and focus on *E*. *coli* frataxin CyaY. Using small-angle X-ray scattering (SAXS), we show that higher iron-to-protein ratios lead to larger oligomeric species, and that oligomerization proceeds in a linear fashion as a results of iron oxidation. Native mass spectrometry and online size-exclusion chromatography combined with SAXS show that a dimer is the most common form of CyaY in the presence of iron at atmospheric conditions. Modeling of the dimer using the SAXS data confirms the earlier proposed head-to-tail packing arrangement of monomers. This packing mode brings several conserved acidic residues into close proximity to each other, creating an environment for metal ion binding and possibly even mineralization. Together with negative-stain electron microscopy, the experiments also show that trimers, tetramers, pentamers, and presumably higher-order oligomers may exist in solution. Nano-differential scanning fluorimetry shows that the oligomers have limited stability and may easily dissociate at elevated temperatures. The factors affecting the possible oligomerization mode are discussed

## Introduction

Frataxin is a highly conserved protein found in both prokaryotes and eukaryotes. It is involved in several central functions in cells, including delivery of iron to different biochemical processes, such as heme synthesis, assembly of iron-sulfur clusters (ISC), storage of surplus iron in conditions of iron overload, and repair of ISC in aconitase [1–5]. Frataxin deficiency in humans, which is a result of low expression levels of the protein, has been linked to the neurodegenerative disease Friedreich’s ataxia [6, 7]. The disease is accompanied by hypertrophic cardiomyopathy, diabetes mellitus, and impairments to mobility and speech ability [4, 8]. High levels of reactive oxygen species (ROS) have also been found in conditions of frataxin deficiency. The most active representative of ROS, the hydroxyl radical OH^•^, was found to cause damage to mitochondrial proteins and DNA through the Fenton reaction, a result of the presence of free ferrous iron in the mitochondrial matrix [9, 10]. The mechanism of function of frataxin and the details of its interaction with other proteins are still not well understood. Although there are differences in the observed function of frataxin from different organisms, one common feature of the protein from bacterial, yeast, and humans appears to be its ability to form higher order oligomeric species. Both *E*. *coli* frataxin (CyaY) and yeast frataxin (Yfh1) have been shown to require iron for oligomer formation, while human frataxin does need iron for oligomerization [2, 11, 12].

One of the primary functions of frataxin from bacteria, yeast and humans is related to its involvement in the ISC assembly machinery [13–18]. A low-resolution SAXS structure of the complex of the components of the ISC assembly machinery with *E*. *coli* frataxin CyaY has been obtained [19]. CyaY from *Bacilus subtilis* has also been shown to interact with ferrochelatase, the terminal enzyme of heme synthesis that catalyzes iron insertion into protoporphyrin IX [20]. In addition, it was shown that *Vibrio cholerae* CyaY binds heme with dissociation constant in the nano-molar range [21], and that heme biding induced oligomerization of the protein. The authors suggest that heme binding to CyaY serves in the regulation of ISC assembly and may facilitate iron transfer to both the ISC assembly complex and to ferrochelatase. In the presence of iron at anaerobic conditions, CyaY was found to form tetrameric structures as well as unspecified larger oligomers [2]. In a later study, the CyaY tetramer was proposed to be the smallest building block for higher-order oligomers, however, the arrangement of tetramers within the larger particles has not been resolved [22].

The involvement of yeast frataxin Yfh1 in various cellular functions has also been demonstrated in a large number of studies. Thus, the iron storage function of Yfh1 was addressed by X-ray absorption spectroscopy (XAS) measurements of iron-loaded multimeric Yfh1, which showed that bound iron was in the ferric state, in the form of a ferrihydrite mineral, a biomineral composed of ferric oxide/hydroxide, which is also found in ferritin [23]. Subsequent EM reconstruction of iron-loaded 24-meric Yfh1 particles confirmed the XAS data and revealed that iron was stored in the form of an iron core, similar to that of ferritin [24]. Recent studies also showed that trimeric Yfh1 could form a complex with ferrochelatase [25]. Frataxin binding to ferrochelatase stimulated the metallation reaction by direct delivery of iron to the enzyme. In addition, large 24-meric Yfh1 particles were shown to form a complex with the ISC scaffold protein Isu1 [17].

Using a combination of electron microscopy (EM) and SAXS, the structures of the oligomers formed by Yfh1 in the presence of iron could be resolved and were shown to be multimers of trimers (hexamers and higher-order oligomers, up to 48-mers), suggesting that a trimer is the smallest building block for the higher-order oligomers [22, 24, 26, 27]. The studies also showed that the poorly conserved N-terminal extension of Yfh1 is essential for trimer and higher-order oligomer stabilization [22, 26, 27].

In contrast to bacterial and yeast frataxin, human frataxin (FXN) has been found to exist in three different isoforms: FXN^42-210^, FXN^56-210^, and FXN^81-210^. These isoforms are formed by the cleavage of the FXN precursor by the mitochondrial processing peptidase after the transfer of the protein to the mitochondrial matrix [28–31]. The two longer isoforms, FXN^42-210^ and FXN^56-210^, have been shown to oligomerize independently of iron during expression in *E*. *coli* [28, 32]. Similarly to yeast Yfh1, FXN^56-210^ was also found to store iron and to build a ferrihydrite mineral core by catalyzing iron oxidation through the ferroxidation reaction [4, 23]. The packing of FXN^42-210^ into large particles was recently revealed by EM single particle reconstruction of the complex of the 24-meric FXN^42-210^ particle in complex with the ISC synthesis machinery proteins NFS1:ISD11:ISCU [16]. Also in this case the poorly conserved N-terminal extension was shown to be essential for stabilization of the oligomers [33, 34]. Moreover, it was demonstrated that the assembly of the particles was reversible, since monomeric FXN^56-210^ obtained after dissociation of the oligomers, could be re-assembled into oligomers after incubation at moderate concentrations (1.5 M) of GdmCl and subsequent dialysis of the denaturant [34]. It cannot be excluded that oligomerization of human frataxin in cells is assisted by chaperones, while the conditions used in the above mentioned work may mimic the presence of chaperones.

The shortest isoform of human frataxin, FXN^81-210^, which due to the absence of a long N-terminal extension may have properties comparable to those of CyaY, is the most abundant form of human frataxin in mitochondria. It is believed to exist predominantly in a monomeric state and to have labile iron binding capacity [28, 35, 36]. However, in a recent study of the effect of iron chelators on frataxin oligomerization we showed that FXN^81-210^ may form oligomers *in vitro* at aerobic conditions in the presence of iron and that the addition of ferric iron chelators deferiprone and DFO stabilizes these oligomers and even stimulates the formation of a larger number of oligomers [37]. Interestingly, in contrast to CyaY oligomers, FXN^81-210^ oligomers were unstable and were found to dissociate after approximately 24 hours [37]. CyaY oligomers on the other hand, dissociated in the presence of chelators, similarly to yeast frataxin Yfh1 oligomers [11].

Although frataxin has a highly conserved amino acid sequence and three-dimensional structure [5], the data so far suggest that the short- and long-forms of the protein from different organisms may use different mechanisms for oligomer stabilization. Establishing the details of these mechanisms will provide a better understanding of frataxin function and the relationships between iron binding and oxidation, iron-induced oligomerization, and the structure of the oligomeric species. It may also help in comprehending the molecular mechanisms of Friedreich’s ataxia, paving the way for the development of new treatments of the disease. From a more general point of view, understanding these mechanisms may provide valuable insights into the way oligomerization within a protein family can be fine-tuned by evolution, creating some functional variety within the family.

Here, iron-induced oligomerization of *E*. *coli* CyaY was studied using a combination of methods, which include size-exclusion chromatography (SEC), small-angle X-ray scattering (SAXS), electron microscopy (EM), native mass spectroscopy (native MS) and nano-differential scanning fluorimetry (nano-DSF). The study addresses the issues of CyaY oligomer formation and the possible architecture and stability of the oligomers.

## Material and methods

### Protein expression and purification

CyaY was purified as described in [38], with the exception of omitting the second-step anion exchange chromatography using Macro-Prep High Q. Protein concentration was determined by measuring the UV absorbance at 280 nm with an extinction coefficient (ε_280 nm_) of 28,990 M^-1^ cm^-1^. The protein was eluted in HN100 buffer (20mM Hepes pH 7.3, 100 mM NaCl).

### SAXS measurements

For studying iron-induced oligomerization, CyaY at a concentration of 1 mg/ml (0.082 mM) in HN100 buffer was incubated with ammonium iron (II) sulphate hexahydrate ((NH_4_)_2_Fe(SO_4_)_2_.6H_2_O) at different Fe^2+^ concentrations that varied from 0.04 mM to 0.82 mM (Fe^2+^: CyaY molar ratios of 1:2, 1:1, 2:1, 4:1, 7:1, and 10:1). The incubation was performed at 30°C for one hour according to published protocols [11], with a final volume of 20 μl. The SAXS measurements were also made at protein concentrations between 0.5 and 10 mg/ml to ensure that no concentration dependence was present. All samples were centrifuged for 10 minutes at 14000 rpm before the experiment. Data were collected at the P12 BioSAXS beamline at EMBL-Hamburg, storage ring PETRA III, at 20°C using a Pilatus 2M detector (1475x1679 pixels) and synchrotron radiation with a wavelength of 0.124 nm. Experimental data were normalized to the transmitted beam intensity, and scattering of the buffer was subtracted. An automatic sample changer was used for a sample volume of 15 μl [39]. No radiation damage was observed in the samples.

For the time-resolved SAXS (TR-SAXS) data, a sample with a 7:1 Fe^2+^ to CyaY ratio was prepared at 3 mg/ml CyaY and a final volume of 650 μl. The sample was then incubated at 30°C in the sample holder at the beamline. The first SAXS measurement was made 5 min after the start of incubation. Subsequent measurements occurred approximately every 3.5 min up to 64.5 min. Two final measurements were then taken after 80 min and 90 min. TR-SAXS data were collected using a Pilatus 1M detector and synchrotron radiation with a wavelength of 0.992 A° at BM29 at ESRF, Gernoble, France [40]. Using the known absolute scattering of water (I_0,abs_(water) = 1.632.10^-2^cm^-1^, at 20°C),) and water measurements (empty capillary for subtraction) we could set our data to absolute scale in order to make molecular weight estimations for the samples [41]. Bovine serum albumin was also measured as a standard for a second source of molecular weight estimation. Standard HN100 buffer was used in buffer measurements. Concentration series of CyaY were prepared by diluting the stock protein solution with the standard elution buffer. A sample changer robot was used to load a 30 μl sample into the measurement capillary, and the SAXS data were collected in flow mode. Automated data collection and initial processing were run using the dedicated beamline software BsxCuBE [40]. Scattering profiles for the buffer were collected before and after each sample measurement, averaged, and subtracted from the sample scattering profile.

Data processing for all SAXS samples was achieved using PRIMUS software [42] and the ATSAS package [43]. I_(0)_ and R_g_ determinations were made using the Guinier approximation. GNOM [44] was used to determine the real-space R_g_, Porod volume V_p_, and the maximum particle dimension D_max_ derived from the pair distribution function p(r).

The OLIGOMER program [42] was used to fit the observed scattering curves using weighted combinations of theoretical form factors from four models. The first model was the monomeric CyaY (PDB entry: 1EW4), while the second and third models were two dimers obtained from modeling with the online SASREF program [45] (*head-to-tail* and *head-to-head* arrangements). The fourth model was a tetramer generated earlier based on a Yfh1 hexamer and used for modeling CyaY SAXS oligomer data [22]. CRYSOL [46] was used to calculate the form factors from the CyaY models.

### SEC of iron-incubated CyaY

CyaY was incubated with ammonium iron (II) sulphate ((NH_4_)_2_Fe(SO_4_)_2_.6H_2_O) at the molar ratio of 7:1 iron to CyaY at 30°C for 1 h before loading on Superdex 75 10/300 GL columns (GE Healthcare). The final concentration for CyaY was 8 mg/ml (0.65 mM) with 4.55 mM Fe^2+^ (a 7:1 ratio of iron to protein). Since the 7:1 iron-to-protein ratio resulted in protein aggregation at protein concentrations higher than 3 mg/ml, 100 μl aliquots of 2 mg/ml CyaY were initially incubated with iron and subsequently concentrated to the required concentration. The sample was always centrifuged for 10 min at 14000 rpm before loading on the columns. The columns were pre-equilibrated with HN100 buffer and operated at a flow rate of 0.5 ml/min at room temperature. Sample elution followed at 280 nm. The size-exclusion chromatography small-angle X-ray scattering (SEC-SAXS) experiment was optimized to obtain a well-separated peak for the iron-induced oligomeric species. The best conditions for the experiment included an iron incubation time of 45 min, followed by filtering the sample using a 100 kDa cut-off membrane (Amicon ^®^ Ultra) to remove aggregates and larger oligomeric particles (> 100 kDa) that could not be separated by the column. The flow-through was then concentrated in a 10 kDa cut-off membrane (Amicon ^®^ Ultra) to 100 μl before loading onto the SEC column.

### Online SEC combined with SAXS (SEC-SAXS)

Online SEC-SAXS data were collected at the BM29 beamline, ESRF, Gernoble, France [40]. The beamline HPLC system was Viscotek GPCmax, Malvern instruments, directly connected to the sample changer. Scattering data were obtained for the 7:1 iron-to-CyaY ratio prepared as described above for the final optimization of the SEC-loaded sample. The only difference was that the sample was prepared using CyaY at a 56 mg/ml concentration in order to get a higher signal to noise ratio. The sample was eluted at a flow rate of 0.5 ml/min and passed through a capillary cell, and data were collected from 3000 scattering frames (one frame per second). The one-dimensional profile for each frame was generated using the EDNA pipeline [47]. The first 200 stable frames, collected before sample elution, were averaged and used for buffer subtraction. The scattering frames for the eluted second peak (oligomeric species peak) were averaged, after which buffer subtraction was performed. Data processing and SAXS parameters estimation was performed as described above. The molecular weight estimation was performed using the porod volume V_p_ [43].

*Ab initio* modeling to fit the SAXS data relied on simulated annealing on a dummy atom set using the DAMMIF program [48] in slow mode with default settings. Twenty independent DAMMIF runs were aligned and averaged using DAMAVER [49], minimizing the normalized spatial discrepancy (NSD) between the models. Models with high NSD were discarded, and the resulting filtered model of the most probable structure was obtained using DAMFILT.

SASREF [45] was used for rigid-body modeling of the scattered data. The crystal structure of the CyaY monomer (PDB entry 1EW4) was used for building a model, which was assumed to be a dimer based on the initial processing of the SAXS data. For modeling of the dimer, the distance between Met1 from the first subunit and Lys65 from the second subunit was restrained to 24 Å [37]. Twenty SASREF models were generated with calculation of χ^2^ for each model. The best model was superimposed on the filtered *ab initio* model using the SUPCOMB program [50], which minimizes the NSD to find the best alignment of both models.

### EM imaging

For EM imaging, samples of CyaY (1 mg/ml) at a 7:1 iron-to-protein ratio were prepared as described above for SAXS experiments. The sample was diluted to 0.4 mg/ml and applied to a 400-mesh carbon copper grid (EMS). The grid was pre-incubated in HN100 for 1 minute before applying the sample. The sample buffer was blotted and washed for 3 seconds in sterile water. Uranyl acetate (1% w/v) was applied to the grid for 30 seconds, and excess stain was blotted directly. The grid was left to dry for 30 minutes before insertion into the sample holder of a Philips CM120 transmission electron microscope equipped with a GATAN GIF 100 energy filter and a GATAN 791 CCD camera (1024x1024 pixels). All images were taken at 55000x magnification. Glow discharge was applied for more uniform spreading of protein sample on the grids.

### Nano-DSF measurements

Nano-DSF measurements were performed using a Prometheus NT.48 instrument (NanoTemper Technologies, GmbH, Germany). The measurements were done for CyaY without added iron and with the same Fe^2+^-to-CyaY ratios and buffer used in the SAXS experiments (1:2, 1:1, 2:1, 4:1, 7:1, and 10:1). The iron-incubated samples had a protein concentration of 1 mg/ml and were prepared in the same way as for the SAXS experiments. Ten μL of each sample was loaded into UV capillaries (NanoTemper Technologies). The temperature gradient was set at 0.5°C/min in a range from 20 to 95°C. Protein unfolding was detected by following the change in tryptophan fluorescence at emission wavelengths of 330 and 350 nm. The ratio between the emission intensities at 350 nm and 330 nm (F350/F330) was used to track the structural changes with increasing temperature. Data analysis was performed using the manufacturer’s software, where T_m_ was calculated using the peaks in the first derivative curve.

### Native MS measurements

All samples were analyzed by using the SYNAPT G2-Si High-Definition Mass Spectrometer (Waters, Manchester, U.K.) [51, 52]. For the analysis, all protein solutions were buffer-exchanged into 200 mM ammonium acetate (pH 7.5) using Micro Bio-Spin chromatography columns (Bio-Rad). Aliquots of ∼2 μL were introduced into the mass spectrometer via nanoflow capillaries under the following conditions: capillary voltage 1.2kV, sampling cone 120V, and source offset 20V. The source temperature was set to 25°C. The collision voltage was adjusted for optimal signal level. Maximum entropy (MaxEnt, Waters) deconvolution was applied to electrospray data to recalculate the gas phase existing masses.

## Results

### Time-resolved SAXS (TR-SAXS) measurements of iron-induced oligomerization

**Fig 1:**
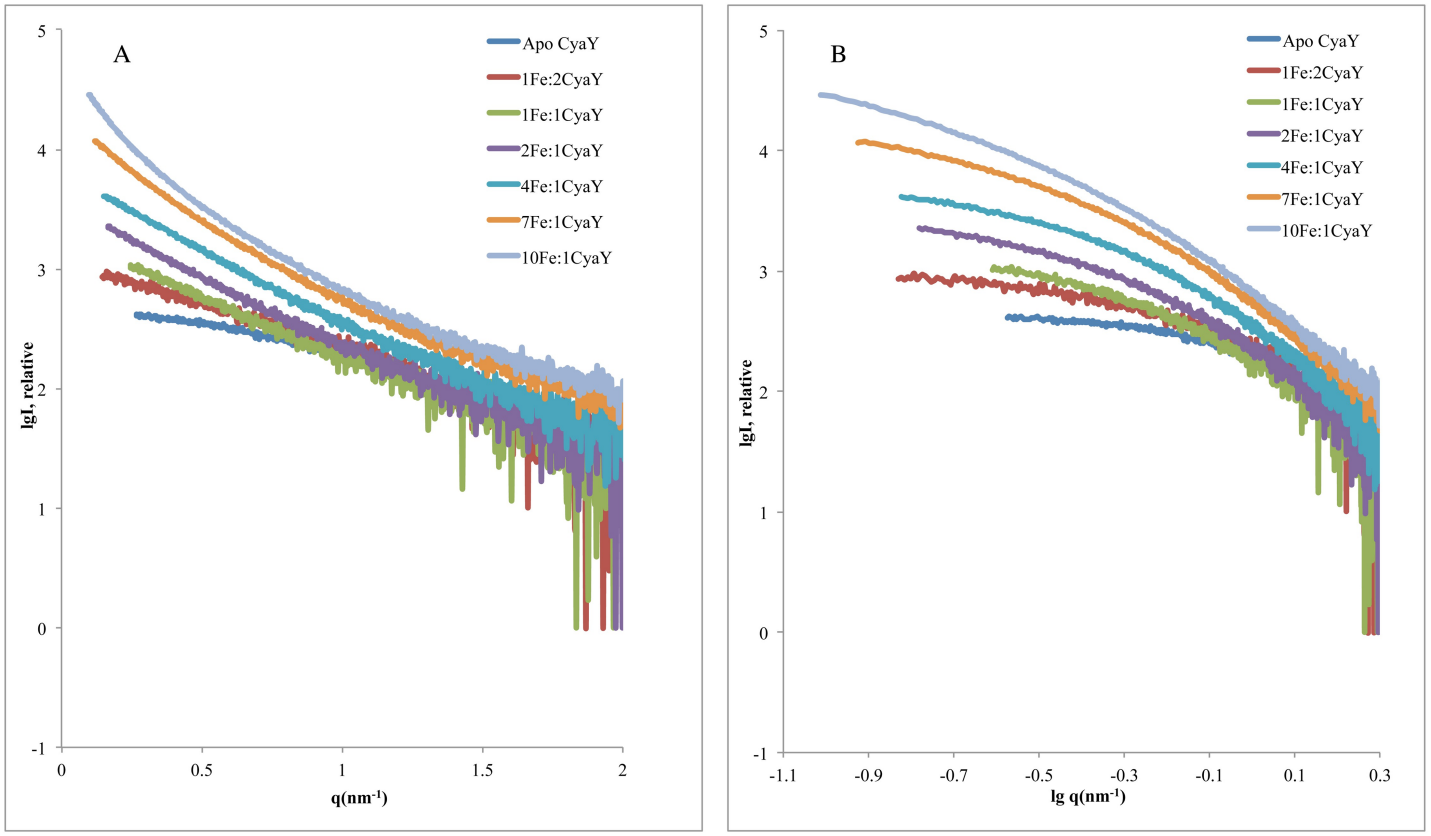
Iron-dependent oligomerization of bacterial frataxin (CyaY). **A)** SAXS scattering profiles for apo- and iron-induced CyaY oligomers at different iron-to-protein ratios. **B)** The double-logarithmic curve for the same SAXS scattering profiles showing apo-CyaY and the oligomeric mixtures of iron-incubated samples.

Earlier studies of iron binding to CyaY showed that it could bind at least two ferrous and up to 25–26 ferric ions/monomer [2]. It was also shown that at the iron-to-protein ratio of 6:1, approximately 50 min of incubation at atmospheric oxygen was required to reach saturation of the absorption curve (absorbance change at 305 nm due to the formation of oxo/hydroxo Fe(III) species was measured in these experiments). This suggests that while CyaY provides partial protection of Fe^2+^ in solution, Fe^3+^ is essential for oligomer formation and stabilization. These results were confirmed in our earlier work [37]. Here, in order to optimize the experimental conditions for time-resolved studies, SAXS measurements of CyaY samples at different iron concentrations were initially run. The SAXS profiles of samples incubated aerobically at six different iron-to-protein molar ratios (1:2, 1:1, 2:1, 4:1, 7:1, and 10:1) are shown in Fig 1A. Table 1 shows the experimental parameters (radius of gyration R_g_, maximum particle dimension D_max_, and Porod volume V_p_) calculated in the absence of added iron and after a gradual increase of the iron-to-protein ratio. In the absence of iron, the data did not show any protein concentration dependence effects (essential to verify in SAXS experiments) and yielded an R_g_ of 1.54 ± 0.01 nm and a D_max_ of 5.29 nm. This value is close to that observed earlier (1.38 ± 0.03 nm) [22]. The slight divengence in R_g_ was found to be due to using two different versions of PRIMUS [42]. When the old data were processed using the same newer version of PRIMUS, the obtained R_g_ and D_max_ values were similar to those obtained in this study. In addition, for iron-incubated samples, differences in R_g_ may result from slight differences in sample preparation, such as small pipetting errors, since very low volumes of iron (between 0.5 to 1 μl) were added to the sample. Finally, variations in the uncontrolled iron oxidation in solution may also affect the results, as the experiments were run aerobically. The estimated V_p_ for apo CyaY was 21.24 nm^3^, which corresponds to an estimated molecular weight of 13.28 kDa. This value is within the normal range of the 10% error for SAXS measurements [53], as the molecular weight of CyaY calculated from the amino acid sequence is 12.23 kDa.

**Table 1:** Radius of gyration (R_g_), maximum size (D_max_), Porod volume (V_p_), and the corresponding molecular weight M_wt_ calculated from the SAXS profiles for CyaY incubated with different iron concentrations.

	R_g_ (nm)	D_max_ (nm)	V_p_ (nm^3^)	M_wt_ (kDa)
**Apo CyaY**	1.54±0.01	5.29	21.24	13.28
**1 Fe: 2 CyaY**	2.95±0.07	10.28	29.79	18.62
**1 Fe: 1 CyaY**	3.08±0.03	11.24	52.5	32.81
**2 Fe: 1 CyaY**	3.99±0.07	15.29	97.98	61.24
**4 Fe: 1 CyaY**	4.68±0.04	16.38	115.14	71.96
**7 Fe: 1 CyaY**	6.60±0.06	23.9	223.85	139.91
**10 Fe: 1 CyaY**	Agg.	Agg.	Agg.	Agg.

SAXS measurements showed increase of the scattering intensity at higher iron-to-protein ratios (Fig 1A) as a result of the systematic buildup of higher oligomeric species or higher volume fractions of these oligomers at increasing iron concentrations. The plateau in the low angle region of the double-logarithmic scattering curves (Fig 1B) for the sample without added iron suggested sample monodispersity. Higher iron concentration resulted in curvature in this region, indicating the presence of an oligomeric fraction (Fig 1B). The estimated R_g_, D_max_, and V_p_ (Table 1) all increased with the increasing Fe^2+^ concentration. D_max_ for the iron-incubated samples ranged from 10.28 nm for the lowest iron concentration up to 23.9 nm for the 7:1 iron-to-protein ratio. The 10:1 sample could not be analyzed due to the presence of aggregated material. It should be noted that since the samples incubated with iron consisted of a mixture of different oligomeric species, the estimated R_g_, D_max_, and V_p_ for these samples reflect an average value for the mixture. The increase in the parameters clearly depends on iron concentration, with higher iron content leading to the formation of higher-order oligomers and/or higher volume fractions of the oligomers.

**Fig 2:**
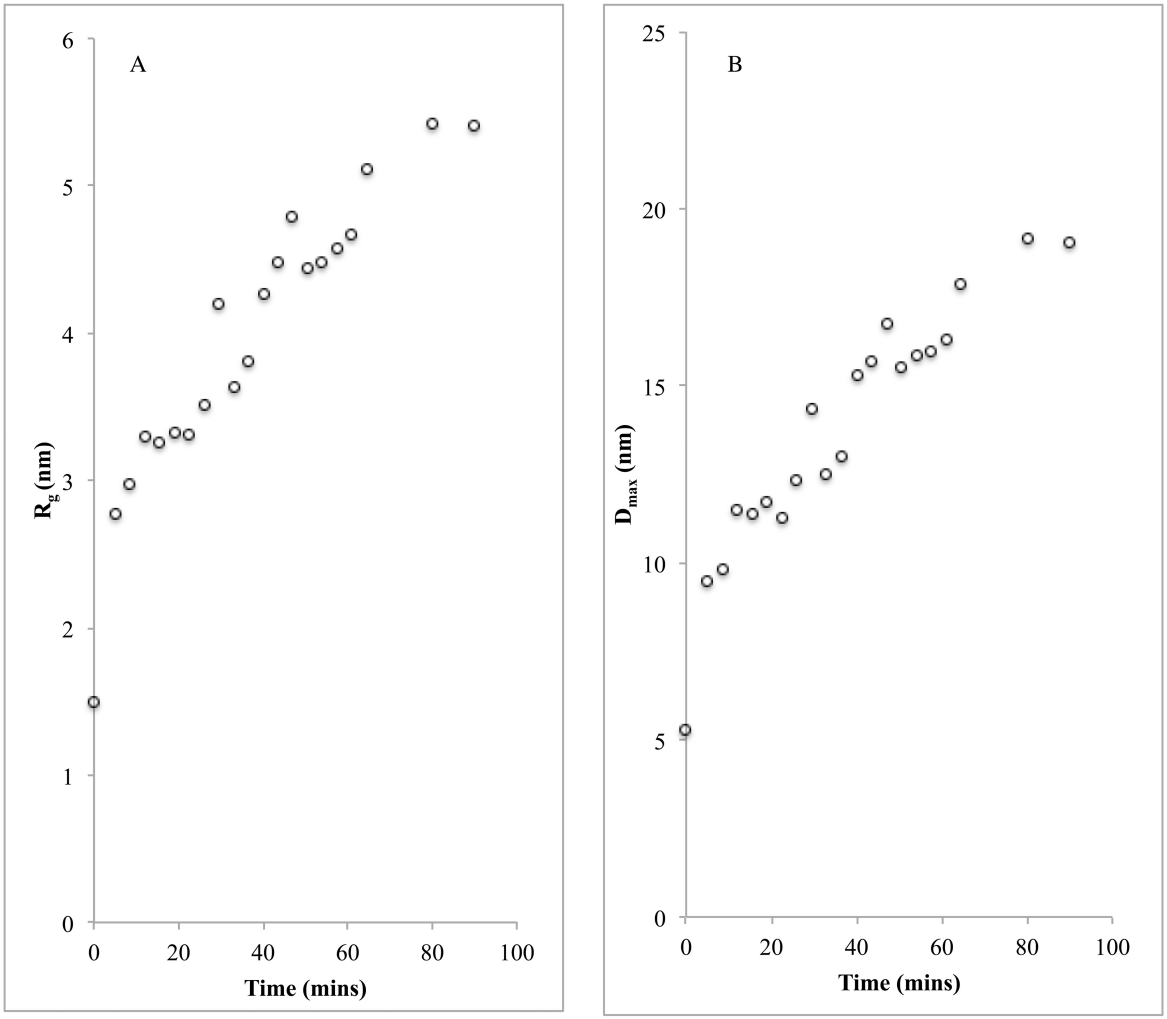
The TR-SAXS parameters for iron-induced CyaY oligomers. **A)** Radius of gyration (R_g_) and **B)** maximum distance (D_max_) plotted against the time of incubation with iron for the sample with 7:1 iron-to-protein ratio.

For the time-resolved study, we followed the course of CyaY oligomerization for one hour at 30°C. The iron-to-protein ratio of 7:1 was chosen to make sure that no protein aggregation occurred in the sample, and using protein concentration of 3 mg/ml. Fe^2+^ was added immediately before the start of the SAXS experiments. The first SAXS measurement began approximately 5 minutes after the start of the incubation. SAXS profiles were then collected every 3.5 min up to 64.5 minutes. Two final profiles were collected at 80 and 90 minutes. R_g_ and D_max_ were calculated separately for each time point (Fig 2). The figure shows a linear increase of the R_g_ and D_max_, indicating that buildup of oligomeric structures takes place parallel to the increase in the degree of iron oxidation. The R_g_ at 0 min was 1.54 ± 0.01 nm, which corresponds to a CyaY monomer (Table 1). Since, as previously mentioned, the protein binds two ferrous and up to 25–26 ferric ions/monomer [2], the gradual increase of R_g_ and D_max_ from 2.78 ± 0.02 nm and 9.49 nm to 4.67 ± 0.03 nm and 16.33 nm, respectively, clearly indicates that Fe^3+^ is required for the formation of large oligomeric structures (Fig 2A and 2B). At 80 min, R_g_ showed an additional small increase to 5.42 ± 0.02 nm, which remained unchanged after 90 min (5.41 ± 0.02 nm), indicating that iron oxidation and the concomitant oligomerization were complete after this period. As noted above, earlier studies showed that after approximately 50 min of incubation at atmospheric oxygen, absorbance of oxo/hydroxo Fe(III) species measured at 305 nm reaches maximum [2], suggesting that some additional slow processes take place before the size of the particles achieves maximum.

### SEC-SAXS measurements

**Fig 3:**
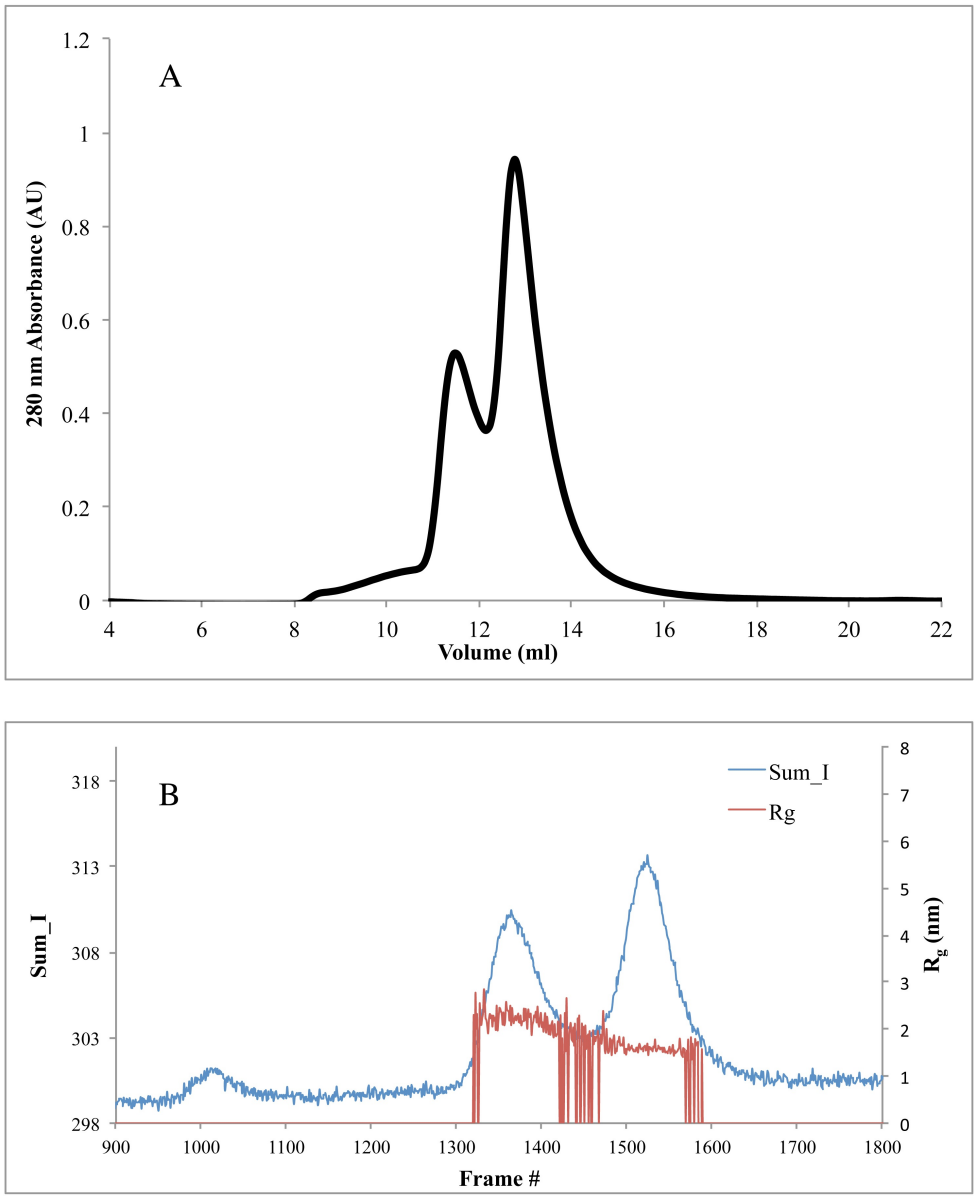
SEC-SAXS for a 7:1 iron-to-CyaY ratio. **A)** Elution profile (normalized absorbance at 280 nm) for the sample with 8 mg/ml CyaY concentration loaded on a Superdex 75 gel filtration column after the elimination of high oligomeric species through 100 kDa concentrators optimized for SEC-SAXS. **B)** SEC-SAXS results for the same sample at protein concentration of 56 mg/ml. Each frame corresponds to 1-second exposure time and is plotted against I_sum (blue) and R_g_ (red). Frames that showed higher stability in R_g_ for the first peak were averaged, and the buffer was subtracted for data processing. The second peak was for monomeric CyaY.

No detailed experimental structural information on monomer packing within CyaY oligomers is available. A tetramer that was generated by analyzing the conserved interactions in Yfh1 hexamers was used earlier for fitting SAXS data and was suggested to be the building block of higher-order oligomers [22]. Here, we used online SEC-SAXS in an attempt to obtain scattering profiles from separate CyaY oligomeric structures. Prior to the SEC-SAXS experiments, the sample was filtered using a 100 kDa concentrator to remove any aggregated material. The iron-to-protein ratio of 7:1 was also chosen for these experiments. Several optimization trials were run, and the best separation was obtained with a Superdex 75 column (Fig 3A) after 45 minutes of incubation at 30°C. A concentrated sample of 56 mg/ml of the protein was used to improve the signal to noise ratio in the SAXS measurements.

The results of the SEC-SAXS experiments are summarized in Fig 3B, where the sum of scattering intensities corresponding to three peaks eluted from the Superdex 75 column is shown. The first small peak corresponds to larger oligomers, which were still present in small fractions after filtering through the 100 kDa concentrator. Unfortunately, this peak had very low scattering intensity, which made it impossible to analyze. The middle peak also corresponds to oligomeric species, and the third corresponds to monomeric CyaY. After frame averaging and buffer subtraction, the SAXS parameters for the middle peak could be estimated. R_g_ was found to be 2.37 ± 0.01 nm, and D_max_ was 8.29 nm. The V_p_ was 36.02 nm^3^, which corresponds to a calculated molecular weight of 22.5 kDa. Taking into account an approximate 10% error in the accuracy of the molecular weight estimation from SAXS measurements [53], the obtained molecular weight most likely corresponds to dimeric CyaY (M.wt = 24.46 kDa). The DAMMIF *ab initio* shape reconstruction program was used to generate models for CyaY dimers. The 20 output models had a mean value for the NSD of 0.658, which is within the normally accepted range for this parameter.

### Rigid-body modeling with SASREF and OLIGOMER

**Fig 4:**
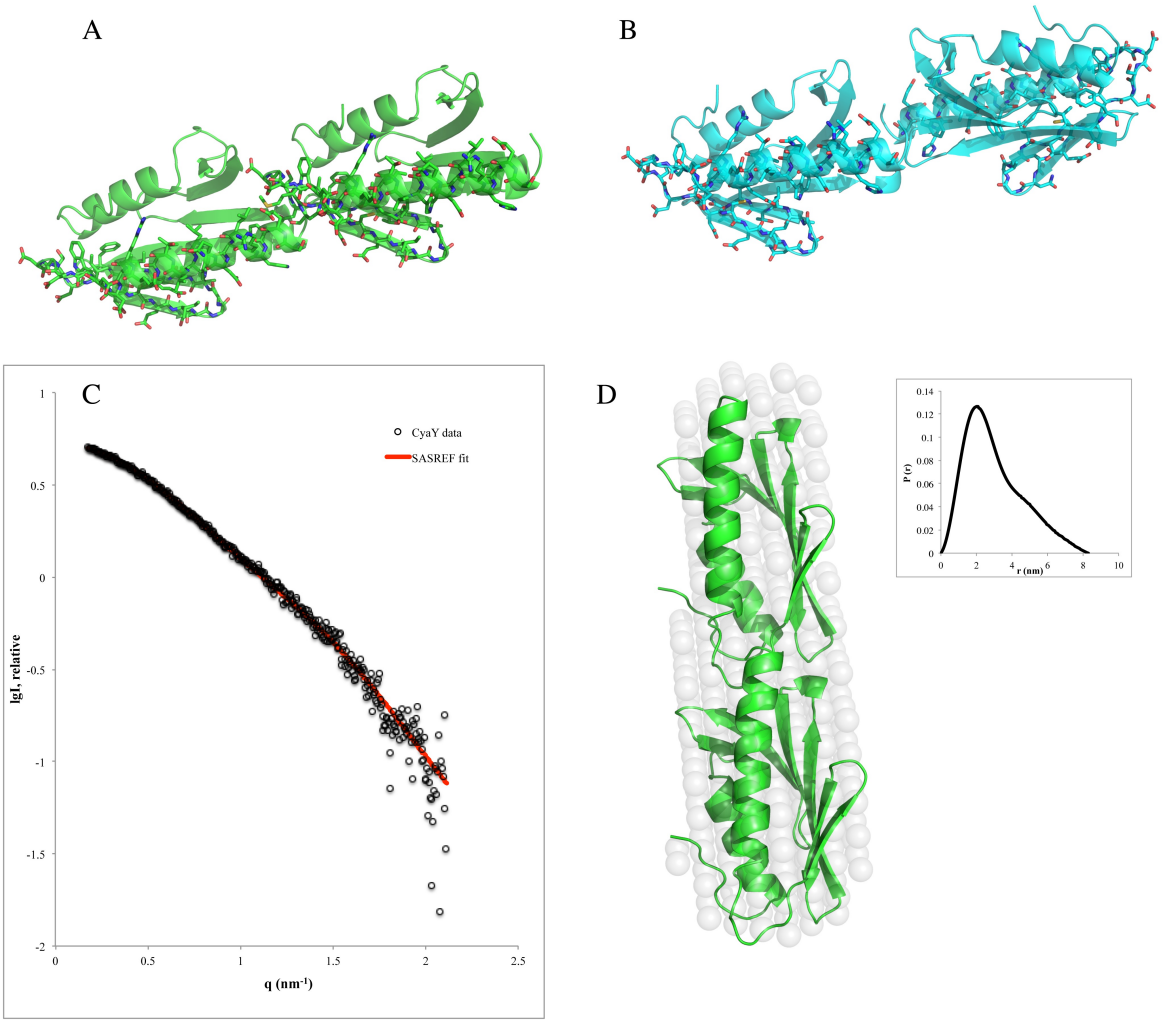
Rigid-body modeling for iron-induced CyaY dimer. The crystallographic structure of monomeric CyaY (PDB entry: 1ew4) was used to generate the CyaY dimer structure using SASREF. Two models were obtained with **A)** *head-to-tail* and **B)** *head-to-head* arrangement of monomers. **C)** SASREF modeling fit of the *head-to-tail* dimer to the SEC-SAXS data. **D)** The *head-to-tail* model docked into the SAXS-filtered *ab initio* model with the corresponding P(r) plot shown in the insert.

SASREF [45] was used for modeling the CyaY dimer from the collected data for the middle peak in SEC-SAX experiment using PDB entry 1EW4. In the modeling, the distance restraint of 24 Å between Met1 of one subunit and Lys65 of the second, which was obtained from cross-linking mass spectrometry (MS) [37], was applied. Two models with a good fit to the experimental data were generated by SASREF (Fig 4). The first model (Fig 4A) had a *head-to-tail* arrangement of monomers, in which the N-terminus (residues 1–3) and the connecting loop between strands β3 and β4 (residues 54–58) of one monomer faced the connecting loop between helix 1 and strand β1 (residues 22–30) and the loop between strands β2 and β3 (residues 43–46) of the second monomer. The second model (Fig 4B) had a *head-to-head* arrangement, where the N-terminus of one monomer (residues 1–3) faced the connecting loop between strand β3 and β4 (residues 56–59) of the second monomer. The N-terminus of the other subunit faced strand β5 (residues 68–74) of the neighboring monomer. It should be noted that cross-linking excluded a *tail-to-tail* arrangement of the monomers. The SASREF fitting of the first model (*head-to-tail*) (Fig 4C) was very good, with χ^2^ = 0.70. Although, the SASREF fitting of the second model (*head-to-head*) was also good, with χ^2^ = 0.72.

The *head-to-tail* arrangement is also supported by the observation that the residues involved in metal binding [54] are brought together (Fig 4A) and may participate in the formation of the ferrihydrite mineral core in conditions of excess of iron. The superposition of the filtered DAMMIF *ab initio* model onto the *head-to-tail* dimer generated by SASREF (Fig 4D) was accomplished with SUPCOMB [50]. The figure shows that the SASREF model is in good agreement with the *ab initio* model, with an NSD value of 0.89.

**Fig 5:**
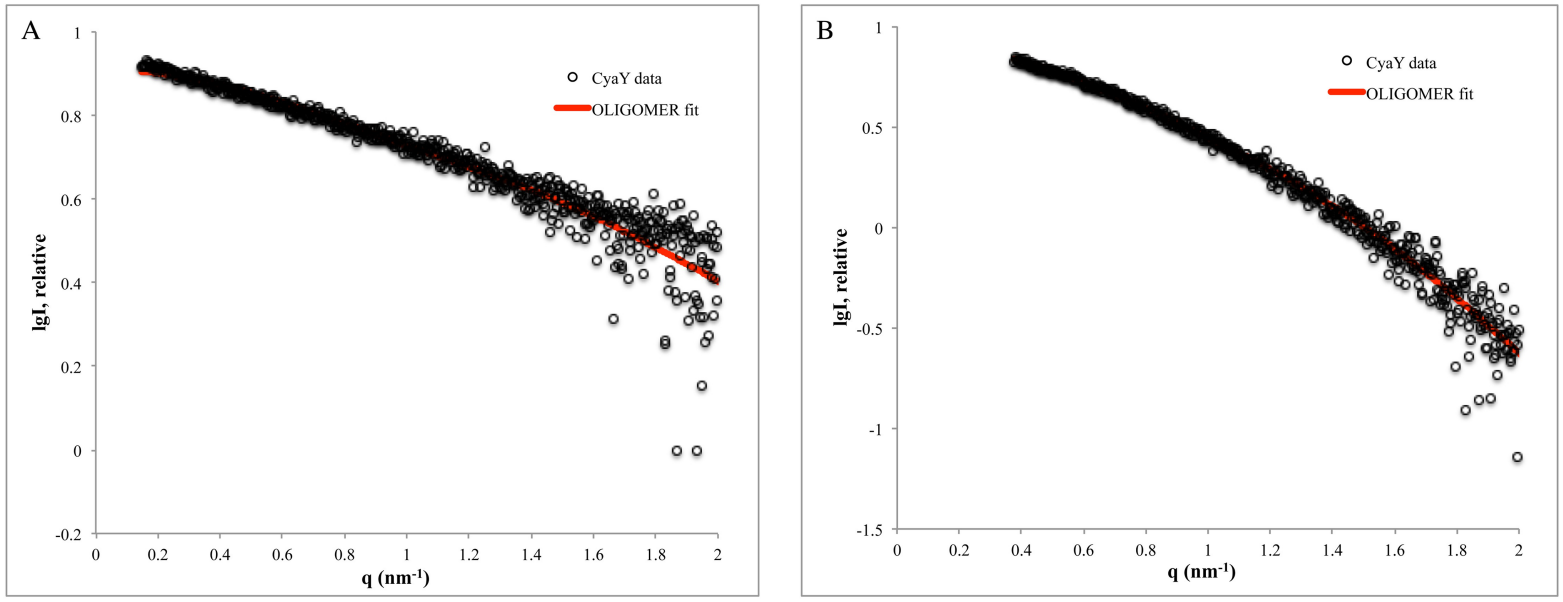
OLIGOMER fitting for the 1:2 iron-to-protein ratio. The experimental SAXS data are represented by circles, while the corresponding fit of OLIGOMER is shown as a red line. **A)** 1:2 iron-to-protein ratio (1 mg/ml of CyaY) and **B)** 1:2 iron-to-protein ratio (3 mg/ml CyaY) as performed in an earlier study [22].

OLIGOMER was used to fit the pool of form factors calculated from generated oligomers to the SAXS data obtained at different iron-to-protein ratios (details can be found in the “Experimental procedure” section). The *head-to-tail* and *head-to-head* dimers from SASREF were also included in the pool. Only the data from the 1:2 iron-to-protein ratio could be modeled with acceptable fitting. The results suggested that 14.5% of CyaY was in the monomeric state and 85.5% was in the dimeric *head-to-tail* state, with χ^2^ of 1.12 (Fig 5A). Using the data obtained earlier at 3 mg/ml protein concentration [22] and the same pool, the modeling suggested that 60.5% of CyaY was in the monomeric state and 39.5% was in the dimeric state, with a very good fit quality (χ^2^ = 0.88) (Fig 5B). Earlier modeling suggested 91% monomeric and 9% tetrameric protein, with χ^2^ = 0.91. In other words, the tetramer option is rejected when a *head-to-tail* dimer could be chosen for fitting the data. As in the previous cases, the modeling at 1:2 iron-to-CyaY ratio preferred the *head-to-tail* arrangement.

### Native MS

In addition to SEC-SAXS, we also used native MS for assessing the oligomerization propensity of CyaY. Native MS is a spectroscopic gas-phase technique, which allows non-covalent protein-protein and protein-ligand complexes to be analyzed. For the analysis, we used a 7:1 iron-to-CyaY ratio sample where the average R_g_, D_max_, and V_p_ values for the mixture identified by SAXS were the highest, suggesting the most abundant content of oligomeric species. The native MS analysis showed a clear signal for the monomer, a weak signal for the dimer, and a yet weaker signal for the trimer (Table 2). The signals for a tetramer and pentamer and up to possibly a hexamer could be detected, but with a lower signal to noise ratio (data not shown). The higher signal intensity obtained for the monomer may be a result of some instability of higher-mass oligomeric species due to buffer exchange or the ionization process (S1 Fig).

**Table 2:** Masses of protein species detected by native mass spectrometry.

Protein complex	MW_cal_ (Da)	MW_measured_ (Da)
CyaY monomer	12231	12229.77±0.72
CyaY dimer	24462	24477.08±1.0
CyaY trimer	36693	36692.96±0.94

### Iron-induced oligomerization studied by EM

**Fig 6:**
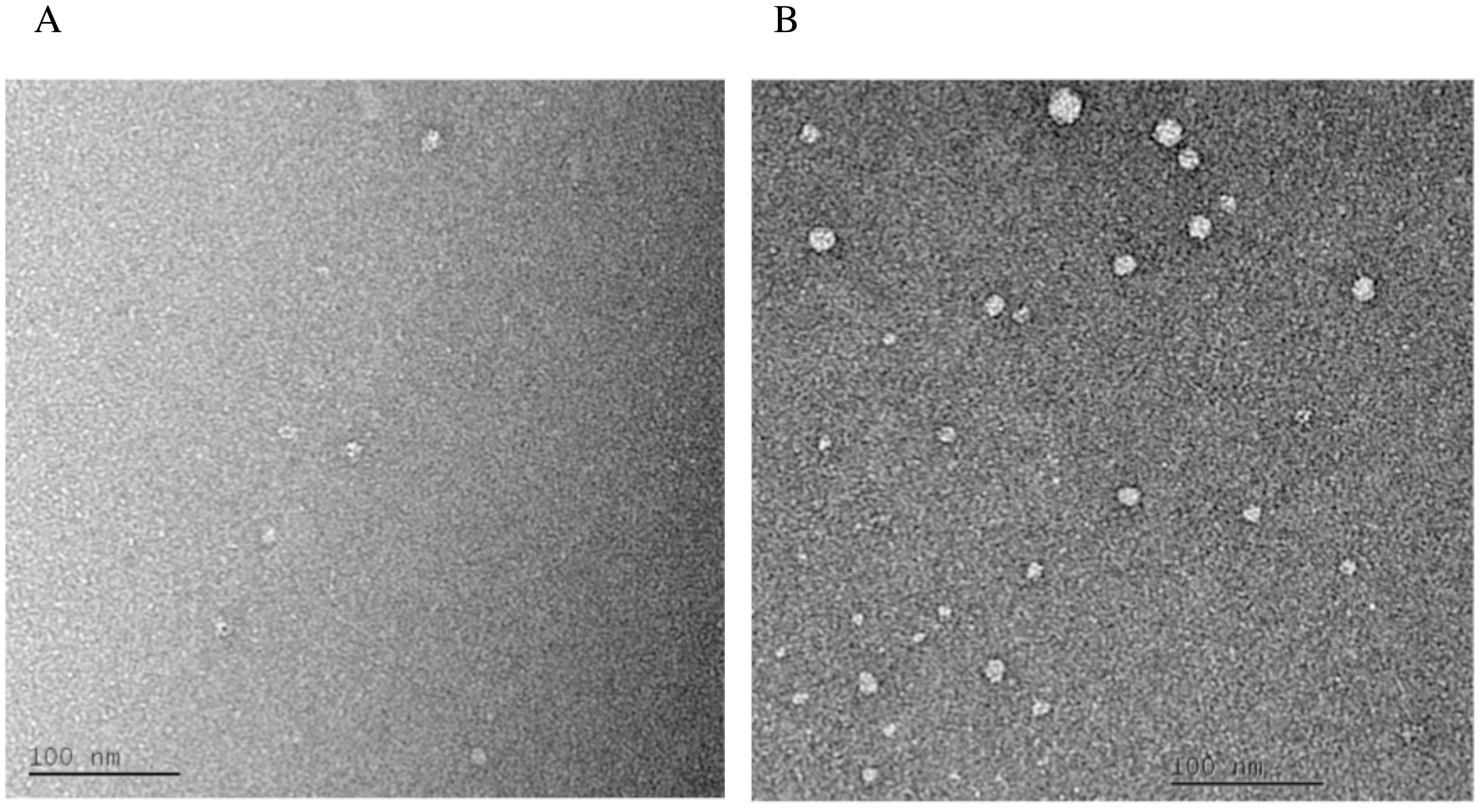
Negatively stained TEM images of iron-induced CyaY oligomers. **A)** & **B)** Iron-incubated sample of CyaY at 7:1 iron-to-protein ratio. Particles of different sizes can be seen in the images (55000x magnification).

CyaY oligomers induced at 7:1 Fe^2+^ to CyaY ratio were also studied using negatively stained EM images. Particles of different sizes, many of which appear to be similar to those observed for FXN^81-210^ iron-induced oligomers [37], may be seen on the transmission electron microscopy (TEM) images (Fig 6). Presumably, these particles correspond to the oligomeric species eluted in the gel filtration experiments. Due to the small number of particles and a large variation in size, it was not possible to perform a detailed analysis using methods of single particle reconstruction at this stage. In addition, the preferred “flat” orientation of the particles excludes any side views from the imaging, which renders a three-dimensional reconstruction impossible.

### Assessing oligomer stability using nano-DSF

**Fig 7:**
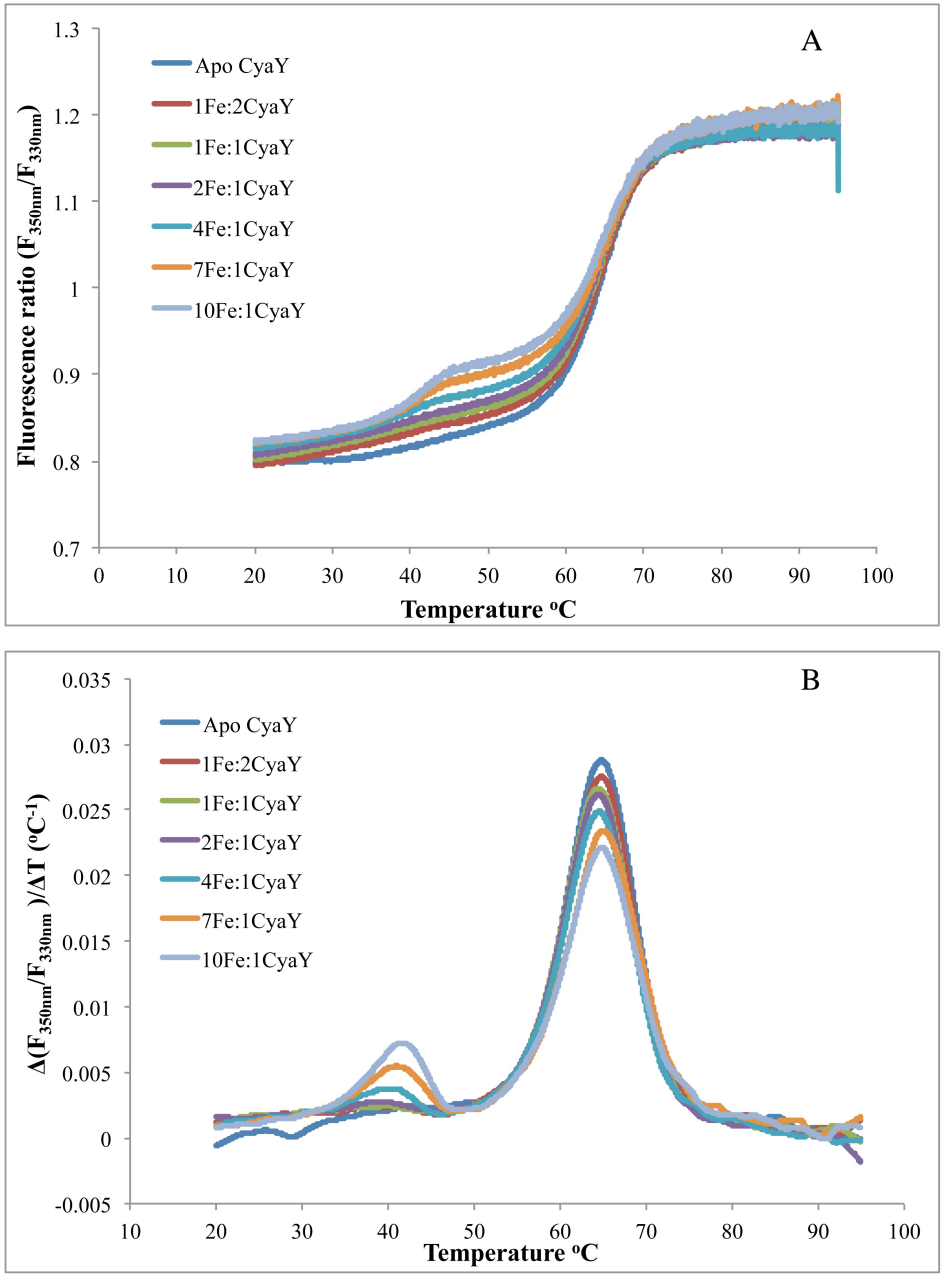
Nano-DSF analysis of iron-induced CyaY oligomer samples. **A)** The F_350nm_/F_330nm_ is plotted against the temperature gradient. **B)** The first derivative for the F_350nm_/F_330nm_ curve against the temperature gradient from which the T_m_ for each sample was derived.

CyaY oligomers have been shown to have higher stability than oligomers of the short variant of human frataxin FXN^81-210^, which were found to dissociate spontaneously into monomers after 24 h [37]. Here, we use nano-DSF for assessing the stability of iron-induced oligomers of CyaY. Nano-DSF is a label-free fluorimetric technique that can determine the thermostability of proteins by following changes in their intrinsic fluorescence. The protein at a concentration of 1 mg/ml was incubated with Fe^2+^ at iron-to-protein ratios of 1:2, 1:1, 2:1, 4:1, 7:1, and 10:1 (the same ratios used in the SAXS studies). For comparison, the protein in the absence of iron was also measured. The melting point (T_m_) for each sample was obtained from the plot of inflection points in the first derivative curve of the emission intensities ratio at 350 nm and 330 nm (F350/330) against the temperature (Fig 7). The results (Table 3) show that iron binding at iron-to-protein ratios 1:2, 1:1, and 2:1 did not affect the T_m_ of the sample (65.5°C, 64.7°C, and 64.4°C, in the absence and presence of iron, respectively). At higher ratios, 4:1, 7:1, and 10:1, there were two inflection points at which two different T_m_ values could be identified—the first was between 64.4°C and 65°C, similar to the values observed for protein without iron and at low iron-to-protein ratios, and the second was substantially lower, ranging between 40°C and 41.5°C. This T_m_ presumably corresponds to dissociation of higher-order oligomeric species.

**Table 3:** Nano-DSF melting points for CyaY incubated at different iron concentrations.

	First T_m_	Second T_m_
**Apo CyaY**	64.5°C	-
**1 Fe: 2 CyaY**	64.7°C	-
**1 Fe: 1 CyaY**	64.4°C	-
**2 Fe: 1 CyaY**	64.5°C	-
**4 Fe: 1 CyaY**	64.5°C	40°C
**7 Fe: 1 CyaY**	65.0°C	41°C
**10 Fe: 1 CyaY**	64.4°C	41.5°C

## Discussion

In this work we continue our explor0ation of the details of iron-induced oligomerization of frataxin and focus on *E*. *col*i CyaY. Using SEC-SAXS and native MS we show that in the presence of iron a dimer is one of the most common multimeric forms of CyaY, although higher order oligomers like trimers, tetramers, pentamers, and perhaps even hexamers may exist in solution. The EM data together with earlier DLS data [37], show that similarly to frataxin from higher organisms, CyaY may form larger oligomeric particles despite the absence of the longer N-terminal extention. However, no experimental structure of any of the higher-order oligomers is available despite the fact that several crystal structures of CyaY with and without bound metals have been determined [55–57]. In an earlier work, in an attempt to model SAXS data, a tetramer of CyaY was constructed after analysis of the packing and the conserved interactions that stabilize yeast frataxin Yfh1 oligomers [22]. The tetramer did fit the SAXS data then; however, as shown here, a dimer with a head-to-tail arrangement (supported by cross-linking MS [37]), fits the SAXS data equally well, albeit with a different percentage distribution of monomers and dimers.

Both the SAXS and EM data show that higher iron concentrations lead to the formation of larger oligomeric species and that oligomerization proceeds in a linear fashion and is directly dependent on iron oxidation. Earlier it was shown that CyaY binds ferrous iron at the stoichiometry of two iron ions/monomer, while ferric iron may bind at a stoichiometry of up to 25 ions/monomer, which are stored as a polynuclear Fe^3+^ hydroxo(oxo) mineral [2]. Taking into account that both yeast and human frataxin have been found to mineralize iron into a ferrihydrite mineral and store it within oligomeric particles [11, 58], it is feasible that ferric iron bound to CyaY would also be mineralized and could contribute to the stability of larger oligomeric structures. The ability of ferric iron chelators to dissociate CyaY oligomers (shown in [37]), suggests that bound iron is easily accessible (for example, for bacterial siderophores) and can be mobilized in bacterial cells in conditions of iron deficiency. This agrees with the observed here relatively low thermostability of large CyaY oligomers, suggesting rather loose packing. It should be noted that the monomer denaturation temperature (around 64.5°C), obtained both in the absence and presence of iron, is in the same range as the temperature obtained in an earlier work in the presence of salts at various concentrations [59]. The slightly higher temperature in our experiment is probably a result of the presence of NaCl in our buffers, which as shown by Adinolfi et al., stabilizes human and yeast frataxin and leads to higher denaturation temperature. However, the above paper does not mention the low-temperature melting point (at around 41°C) observed in our nano-DSF study. The reason is most probably related to the different methods used—while we use DSF, Adinolfi et al. used CD spectroscopy, which registers changes in the secondary structure content of the protein during denaturation. Dissociation of oligomers, unless it involves denaturation of the protein, cannot be observed with CD spectroscopy.

**Fig 8:**
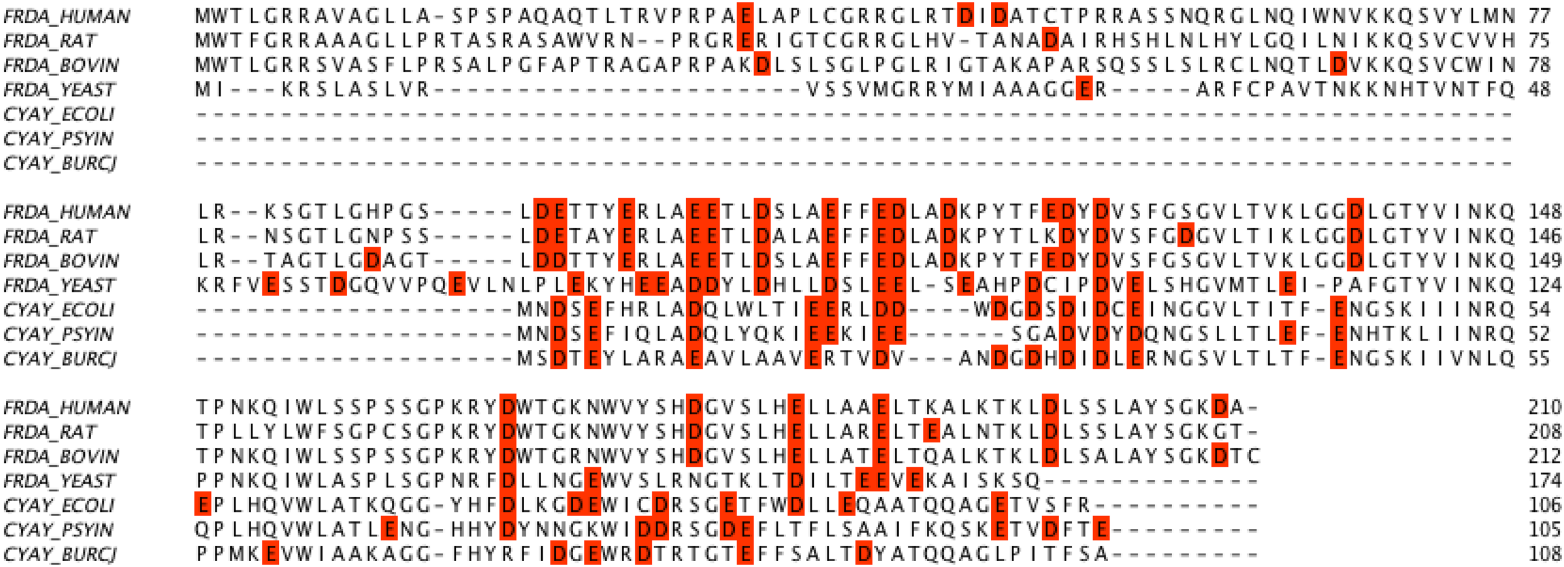
Alignment of CyaY sequences. The acidic residues in the sequence are boxed in red for clarity. CYAY_ECOLI–*E*. *coli*; CYAY_PSYIN—*Psychromonas ingrahamii*; CYAY_BURCJ—*Burkholderia cenocepacia*.

The dimer model of CyaY in the presence of iron was deduced from our current SAXS data, but it also agrees with the earlier suggested packing of monomers, which was based on cross-linking mass spectrometry and docking studies and that also suggested a head-to-tail arrangement [37]. This arrangement of monomers of CyaY is different from that found in yeast and human frataxin oligomers, in which a head-to-head arrangement of monomers within trimers is prevalent [16, 26]. The head-to-head arrangement allows for the N-terminal extension of the protein to interact with neighboring monomers, thus stabilizing the trimer. The stability of the CyaY dimer, on the other hand, was suggested to be a result of the higher number of acidic residues at the monomer-monomer interface [37]. Three potential iron binding sites could be distinguished at the interface. The sites are built up by residues D3, H7, H58 and H70 from one monomer, and E19, D22, D23, D25, D29 and E44 from the second (Fig 8).

Residues D3, E19, D23 D55 and H58 have been found to interact with metals in crystals [57]. The acidic residues, by providing binding sites for iron, could stimulate initial mineralization and dimer stabilization. As our results show, larger particles will be formed only at higher iron-to-protein ratios and after the oxidation of the iron. Human and yeast frataxin, on the other hand, primarily rely on the flexible N-terminal extension for initial oligomer stabilization, but also for the stabilization of higher order oligomers [16, 22, 26, 27]. This may explain the low stability of FXN^81-210^ oligomers, as compared to CyaY oligomers [37].

**Fig 9:**
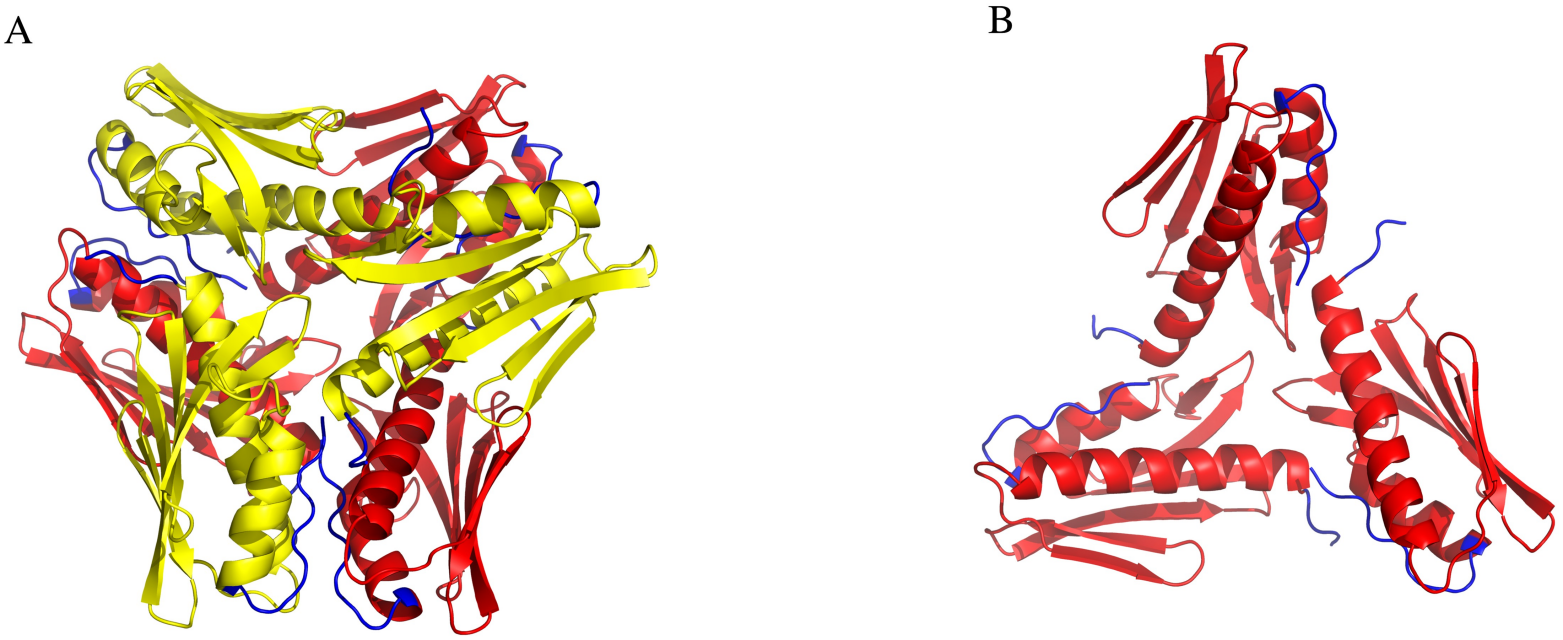
*Burkholderia cenocepacia* CyaY monomer assembly in the crystals (PDB entry: 4JPD). **A)** The hexamer is built up by two trimers packed against each other around the threefold symmetry axis of the crystal. One trimer layer is shown in yellow and the other in red. The N- and C-termini of the subunits are labeled in blue. **B)** One of the trimer layers of the hexamer showing the arrangement of the monomers.

Most of the CyaY residues shown earlier to be involved in iron binding belong to one of the conserved and most prominent features of the frataxin structure—a cluster of acidic residues, which extends from helix 1 (residues 94–114, 76–88 and 3–22 in human, yeast Yfh1 and *E*. *coli* CyaY sequences, respectively) to the first β-strand of the central β-sheet (residues 124–128, 92–94, and 26–28 in human, yeast and *E*. *coli* sequences, respectively). In the human protein, for example, there are in total 13 acidic residues in the cluster, 8 of which are located in helix 1. In yeast and *E*. *coli* 5 and 7 acidic residues (of the total of 13 and 11, in Yfh1 and *E*. *coli* CyaY, respectively) are found in helix 1. However, only 3 of the cluster residues have conserved position across species (Fig 8), while the positions of the others are variable. It appears that this variability together with slight differences in the structure may modulate the interactions between subunits within oligomeric frataxin structures. Thus, in the case of CyaY this variability results in clustering the acidic residues in a way, that brings them close to the area of monomer-monomer interactions. Interestingly, CyaY from *Burkholderia cenocepacia* (PDB entry: 4JPD) has only 5 acidic residues in helix 1, which is similar to the yeast structure. Analysis of the packing of the molecules within the crystals of *B*. *cenocepacia* CyaY shows that the monomers are arranged as two trimers packed against each other around a threefold symmetry axis (Fig 9A).

**Fig 10:**
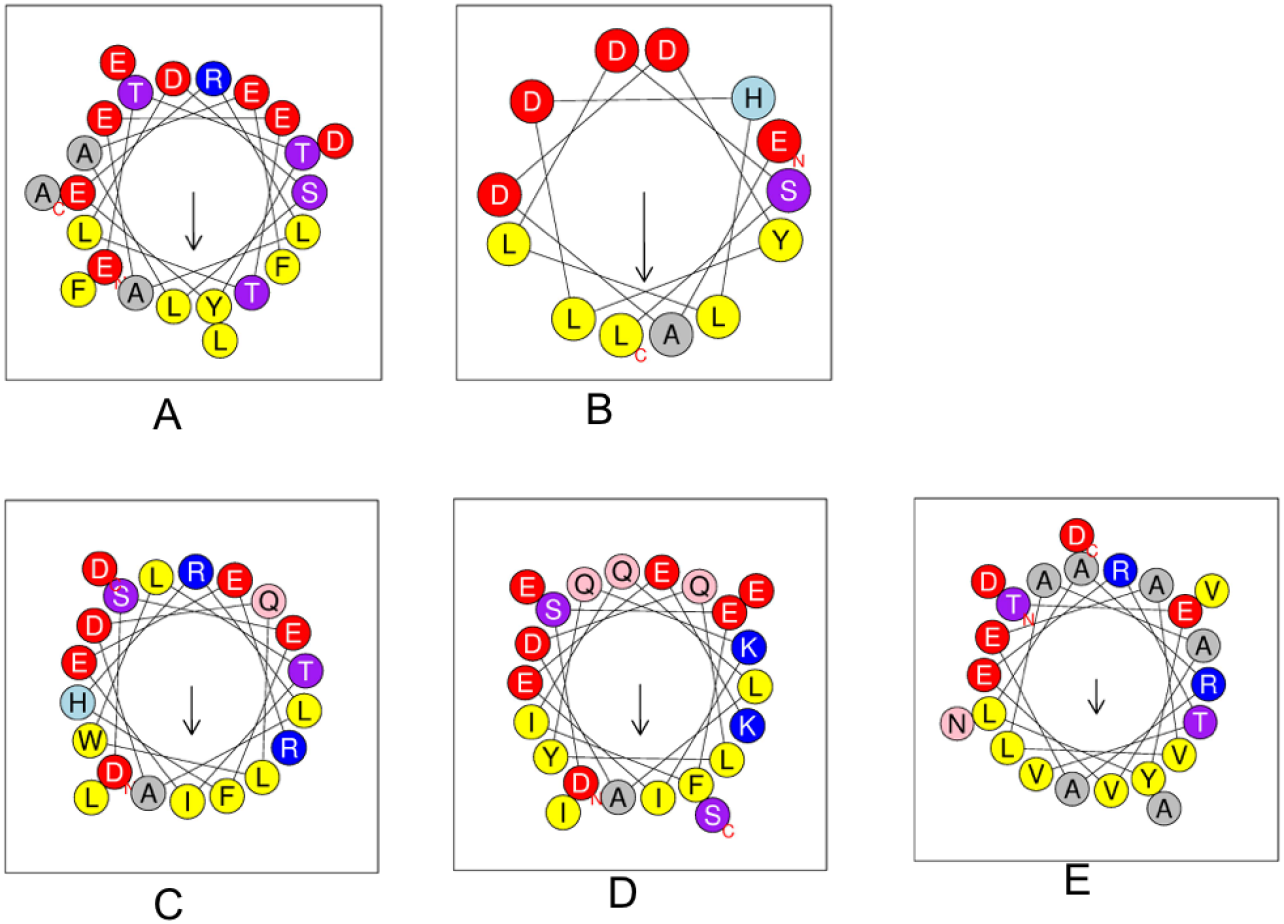
Helical wheel representation of helix 1 of five frataxin structure. (A) Human frataxin; residues (94–114, PDB entry 1EKG) (B) Yeast frataxin Yfh1 (residues 76–88; PDB entry 3OEQ) (C) *E*. *coli* CyaY; (residues 3–22; PDB entry 1EW4) (D) *Psychromonas ingrahamii* CyaY (residues 3–24; PDB entry 4HS5) (E) *Burkholderia cenocepacia* CyaY (residues 4–26; PDB entry 4JPD). Acidic residues are shown as red filled circles, basic residues as blue, polar in magenta and hydrophobic in yellow. The letters represent the amino acids in one-letter code. Figure prepared using the HeliQuest server [60].

As seen in Fig 9B, the packing of the monomers in this case is different from what is observed in CyaY and somewhat reminiscent of the packing found in yeast and human frataxin oligomers. A comparison of acidic residue distribution along helix 1 using helical wheel representation shows that for *B*. *cenocepacia* it is more similar to that of helix 1 from Yfh1 (which also prefers a trimeric arrangement) (Fig 10), while *E*. *coli* and *Psychromonas ingrahamii* CyaY resemble human frataxin to a larger degree. This probably explains the differences in the packing and suggests that frataxin from different bacteria may show different oligomerization modes. It is also interesting to see that the amphipathic character of helix 1 is highly conserved in all known structures (Fig 10). Further studies may shed better light on the role of the different acidic residues in the stabilization of the monomer-monomer interaction surfaces.

Due to the low signal/noise ratio of the higher oligomeric species, it was difficult to detect their presence reliably in native MS and SEC-SAXS studies. Only monomers, dimers, and trimers could be reliably detected. The low volume fractions of the larger oligomeric species may be a result of lower stability, as shown by nano-DSF. The presumed dissociation of oligomers may also be a result of the buffer conditions used, which included acetate, a compound known to chelate iron. Further optimization of the experiments may be required in this case. However, the data clearly demonstrated that native MS can be used in the study of this system. It should be noted that the trimers observed in native MS could also be identified using the SDS-PAGE of crosslinked samples of CyaY and FXN^81-210^ [37].

Although *in vitro* results, specially when protein-protein interactions and protein oligomerization are concerned, are not always easy to verify directly in living cells, it still should be possible to link the findings to processes observed *in vivo*. As noted in the introduction, one of the primary functions of CyaY is related to its involvement in the ISC assembly machinery [19, 61], but also in heme synthesis [20], and presumably in the regulation of ISC assembly [21]. In addition, heme biding appears to induce oligomerization of the protein, although the type of the oligomers is not known. While the most obvious aim of iron binding and oligomerization could be detoxification of surplus iron in conditions of iron overload, as noted earlier, oligomerization may also be involved in the regulation of ISC assembly. Taken into account that only recently the heme binding properties of CyaY became known, it appears that further research is still required to elucidate the details of the function of this protein in cellular iron homeostasis.

## Supporting information

S1 Fig Top-down mass spectrometry analysis of the CyaY oligomeric states. For the analysis, a 7:1 iron-to-CyaY ratio sample was used. Numbers indicate detected ionization states of CyaY.
(PDF)
