# Supplementary material for: SAXS and stability studies of iron-induced oligomers of bacterial frataxin CyaY
Source: PLoS One. 2017 Sep 20;12(9):e0184961. doi: 10.1371/journal.pone.0184961 (PMC5607177; doi:10.1371/journal.pone.0184961)

Supporting information

S1 Fig. Top-down mass spectrometry analysis of the CyaY oligomeric states. For the analysis, a 7:1 iron-to-CyaY ratio sample was used. Numbers indicate detected ionization states of CyaY.

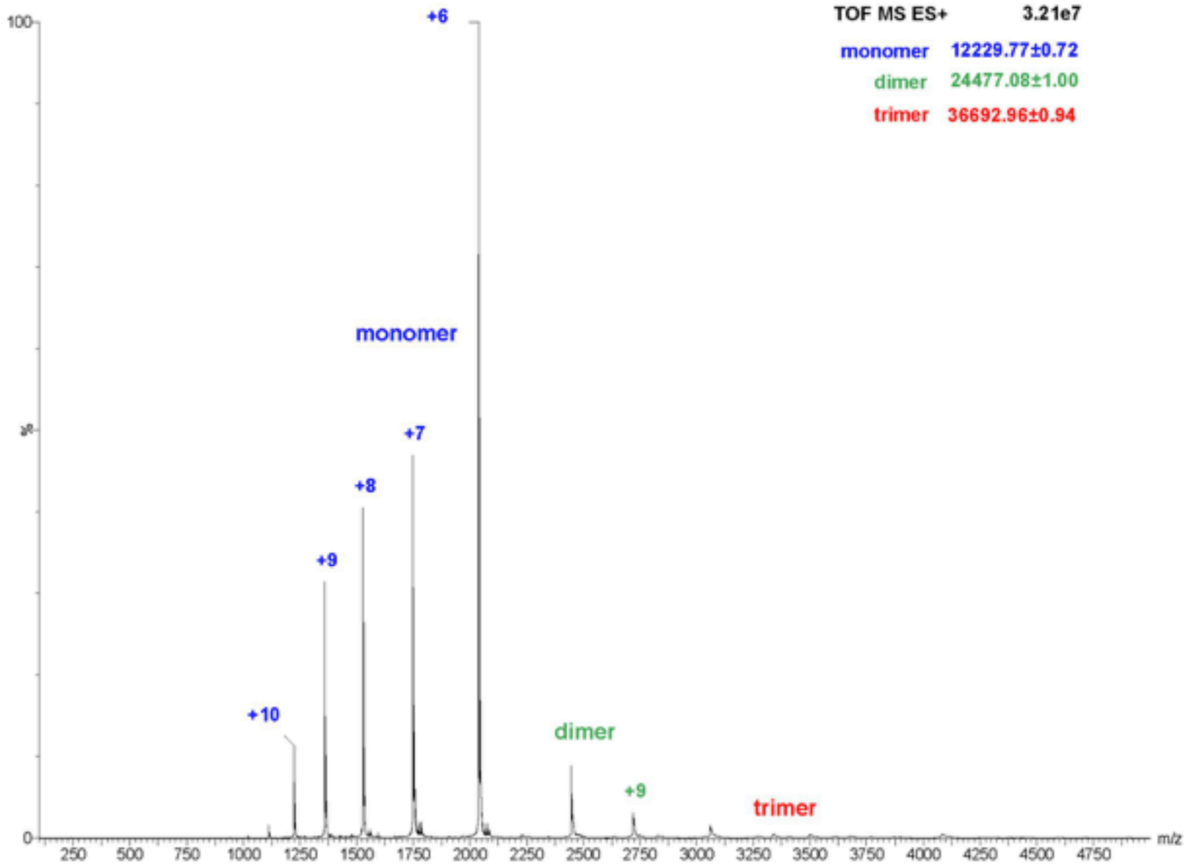

Supplement: S1 Fig — For the analysis, a 7:1 iron-to-CyaY ratio sample was used. Numbers indicate detected ionization states of CyaY. (PDF) [file pone.0184961.s001.pdf]
